# A Review on Bacteriorhodopsin-Based Bioelectronic Devices

**DOI:** 10.3390/s18051368

**Published:** 2018-04-27

**Authors:** Yu-Tao Li, Ye Tian, He Tian, Tao Tu, Guang-Yang Gou, Qian Wang, Yan-Cong Qiao, Yi Yang, Tian-Ling Ren

**Affiliations:** 1 Institute of Microelectronics, Tsinghua University, Beijing 100084, China; yt-li15@mails.tsinghua.edu.cn (Y.-T.L.); tian-y17@mails.tsinghua.edu.cn (Y.T.); tt16@mails.tsinghua.edu.cn (T.T.); guangyangcsu@163.com (G.-Y.G.); wang-q15@mails.tsinghua.edu.cn (Q.W.); qyc16@mails.tsinghua.edu.cn (Y.-C.Q.); 2 Tsinghua National Laboratory for Information Science and Technology (TNList), Tsinghua University, Beijing 100084, China

**Keywords:** bacteriorhodopsin, biohybrid electronic devices, photocycle intermediates, proton pump function, photochemical, photoelectric

## Abstract

Bacteriorhodopsin protein extracted from *Halobacterium salinarum* is widely used in many biohybrid electronic devices and forms a research subject known as bioelectronics, which merges biology with electronic technique. The specific molecule structure and components of bR lead to its unique photocycle characteristic, which consists of several intermediates (bR, K, L, M, N, and O) and results in proton pump function. In this review, working principles and properties of bacteriorhodopsin are briefly introduced, as well as bR layer preparation method. After that, different bR-based devices divided into photochemical and photoelectric applications are shown. Finally, outlook and conclusions are drawn to inspire new design of high-performance bR-based biohybrid electronic devices.

## 1. Introduction

Bioelectronics is an emerging discipline formed by the cross-penetration of biology and electronic information science [1,2,3,4,5,6,7]. Traditional bioelectronics devices are mainly focused on the detection and analysis of biological signal through electrical methods, which rarely involve the incorporation of biological materials into electronic device [8,9,10]. In the early 1970s, the wild-type *Halobacterium salinarum* (*H. salinarum*) as the source of bacteriorhodopsin (bR) was found in the salt marsh archaeon with low oxygen tension, and opened the door of bR-based bioelectronic devices by using the proton pump function and specific photocycle intermediates of bR. [11,12,13,14,15,16,17]

**Figure 1:**
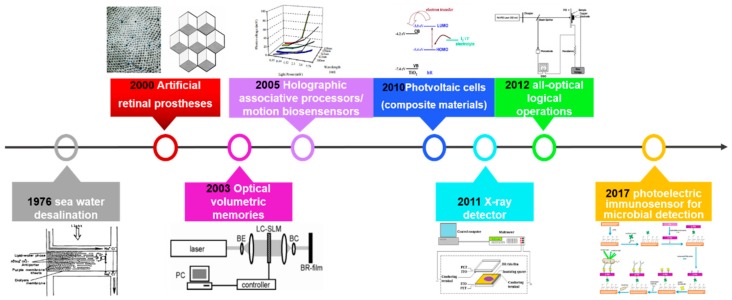
The Roadmap of bR-based bioelectronics applications.

Figure 1 shows the roadmap of bR-based bioelectronics applications since the discovery of bR protein which reveals the development of bR application. In the early stage of bR research, researchers focused more on the structure and biochemical reaction process of bR than its application, and sea water desalination is one of the application directions [18]. Afterwards, bR related artificial retinal prostheses were firstly proposed for the similar optical properties between bR and retinal in the retina [19]. Then, bR was applied in the field of computer systems and sensors, leading to production of optical volumetric memories [20,21,22], holographic associative processors [23,24] and motion biosensors [25,26,27] in the early 2000s. After that, bR was used to compound with a variety of photoelectric materials, which resulted in the generation of photovoltaic cells based on different composite material systems [28,29,30,31,32,33,34]. In 2011, the spectral range of bR related detectors was broadened. Ahmadi et al. developed an X-ray sensor based on bR with a radius of r = 3 mm as the sensing area on a flexible substrate, thus bR’s sensing application is extended to the field of radiation detection [35]. Besides, the photovoltaic response of bR molecules was also utilized in designs of optoelectronic logic gates later [36]. Recently, a photoelectric immunosensor using purple membranes (PM) as the transducer is demonstrated for direct and label-free microbial detection which expands the application field of bR [37]. This review will mainly focus on the photo property application of bR molecules and divide them into two parts, namely photochemical and photoelectric applications, which are introduced in detail in the following article.

**Figure 2:**
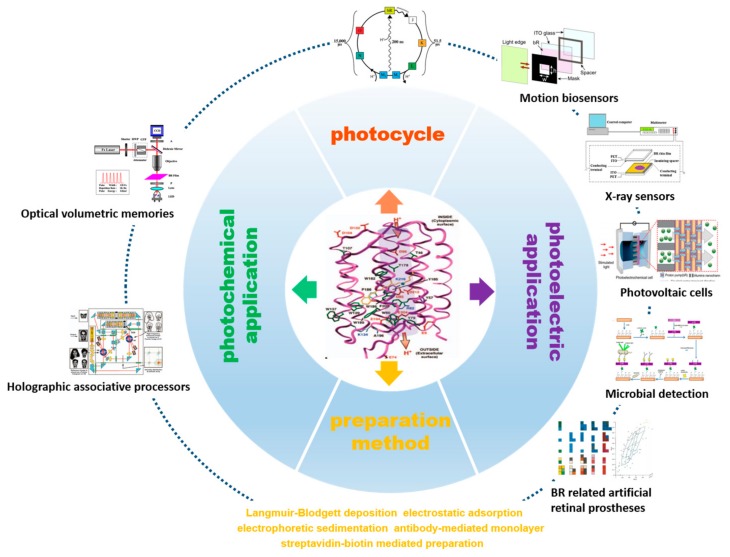
An overview of bR-based bioelectronic devices showing the photocycle, preparation method, and photochemical and photoelectric applications.

In this review, we provide a summary of properties of bR and recent progress of bacteriorhodopsin-based bioelectronic devices. As shown in Figure 2, the photocycle property of bR is the key driving force to develop various bioelectronics applications, which consists of several intermediates and specific branched photocycle. Besides, highly oriented purple membrane can be acquired through different preparation methods, which provides strong support for the fabrication of bR-based electronic devices [38,39,40]. Specifically, these electronic devices are the integration of bR with photoelectric sensing systems and can be developed into two parts: photochemical and photoelectric applications. These works may inspire new design of high-performance bR-based bioelectronic devices and their applications in bioelectronics systems.

## 2. Working Principle and Properties of Bacteriorhodopsin

### 2.1. General Structure and Operation Principles of bR

**Figure 3:**
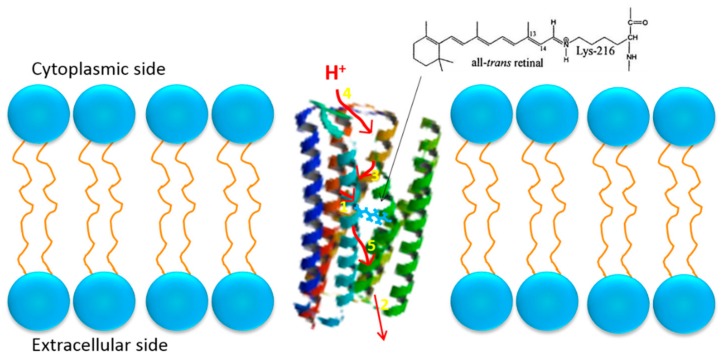
Schematic structure of bR molecule which contains seven α-helices. The red arrows and the corresponding numbers (in yellow) indicate the path of the transport of a proton from the cytoplasmic to extracellular side of the membrane. The insert on the top right corner is the molecular formula of lysine-216. Reproduced from [51].

Extracted from *H. salinarum*, bR molecule is made up of 248 amino acid residues in a polypeptide chain, which consists of seven transmembrane α-helices (shown in Figure 3) [41,42,43,44,45]. Each bR molecule contains a chromophore named retinal, which is covalently linked to the amino group of lysine-216 in the G helix via a protonated Schiff base [46,47,48]. The Schiff base is located in the center of a cavity enclosed by seven transmembrane helices, which effectively divides the proton channel into two partitions: the extracellular and cytoplasmic half-channels [42,44,49]. The extracellular region and cytoplasmic region mostly contains charged residues (e.g., Asp 85) or hydrophobic residues, which serves as the proton acceptor or proton donor to Schiff base, respectively [50,51].

Apart from the discovery of bR from *H. salinarum*, three kinds of membrane proteins containing retinaldehyde were found in halophilic bacteria subsequently: halor-rhodopsin (hR), slow-rhodopsin (or sensory-rhodopsin I, sR-I), and phobo-rhodopsin (or sensory-rhodopsin II, sR-II) [52,53,54]. These four membrane proteins in *H. salinarum* have many similarities in structure and function, which form the family of rhodopsin in *H. salinarum*. Table 1 summaries the similarities and differences of these four proteins. However, each bacterium has only about 20,000 hR molecules, 5000 SR-I and 5000 sR-II molecules, which are hardly extractable compared with bR. Because of the low content, these three proteins were found later, and the related study was far less detailed. Thus, the review on bR-based applications is valuable and necessary.

**Figure 4:**
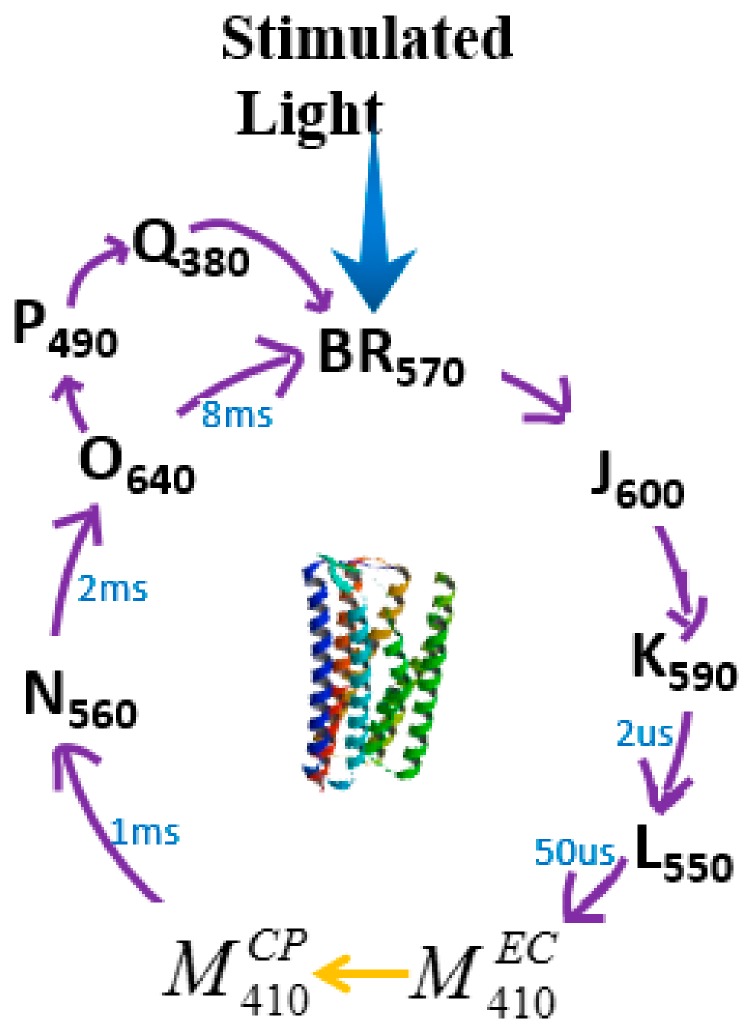
The schematic model of bR photocycle with several intermediates. The subscript of each intermediate stands for their absorption maxima.

Two kinds of retinal chromophores exist in bR naturally: all-trans and 13-cis [55]. In the dark condition, the retinal chromophore in moiety of bR population is all-trans, which is bound to protein via protonated Schiff base linkage to lysine-216 [56]. Under light stimulating, the all-trans retinal is converted to the 13-cis retinal and the photoisomerization of the retinal chromophores provokes photochemical reaction in bR proteins [56,57]. Although the complex process involves several intermediate states (bR, K, L, M, N, and O) with distinct spectral absorption maxima called the photocycle of bR, bR usually spends approximately 15 ms on translocating a proton from the cytoplasmic side to the extracellular side of the membrane, thus accomplishing the photoelectric energy conversion (shown in Figure 4) [15,58,59,60,61,62,63,64,65]. During the photocycle, not all of the bR photo-intermediates can go back to the ground bR state through illumination. Only some intermediates can either thermally relax to the next intermediate or switch directly back to bR state excited by photons with appropriate wavelength [60,66]. The irreversible transition from M^EC^ to M^CP^ and the branched photocycle are the two unique features of bR photocycle [67,68,69].

Firstly, in the irreversible transfer process from the M^EC^ to M^CP^, the nitrogen in the Schiff base can only enter the cytoplasmic side of the half-channel, but it cannot enter to the extracellular side again. That is just the origin of vector proton transfer through cell membranes [67,68]. This verctorial proton transfer property is usually used in the photoelectric applications [70]. Besides this, branched photocycle is another unique feature for bR proteins. During the branch photocycle, the last and most red-shifted intermediate O state will adsorb another red photon, and then it will photoisomerize to P and Q states which contain a 9-cis retinal [71,72]. The Q state is the only thermally stable photoproduct with the lifetime up to 7–12 years at ambient temperature, which is critical in page-oriented optical memory and volume-holographic applications [73,74].

### 2.2. Methods of bR Layer Preparation

**Figure 5:**
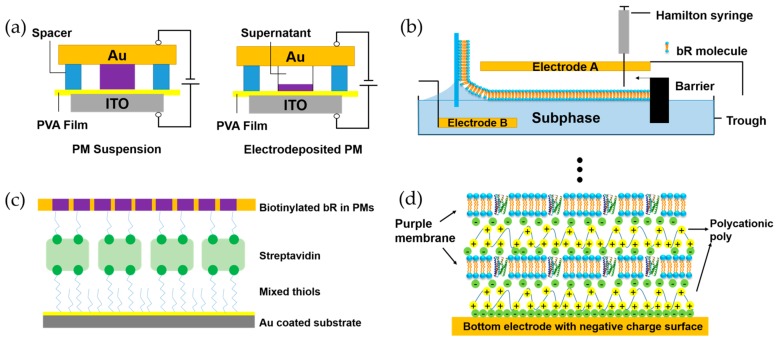
The Schematic structure of different bR layer preparation methods: (**a**) Electrophoretic sedimentation of PM onto a PVA film. +4 V voltage was applied to the PVA film for 5–10 min and PM patch was deposited on the bottom electrode with supernatant appeared in the upper layer. Reproduced from [91]. (**b**) Langmuir–Blodgett deposition of bR onto a solid substrate. The direction of the applied electric field can control the bR orientation. (**c**) Simplified structure of stable PM deposited by biotin-streptavidin mediated monolayer technique. Reproduced from [92]. (**d**) Schematic structure of PDAC/bR multilayers on negative charge surface. Reproduced from [98].

Oriented bR layer is needed in the integrated hybrid devices to generate sufficient photoelectric signal [75,76]. The goal of bR orientation technology is to orient the protein effectively and prevent the purple membrane (PM) from denaturation. Different technologies have been introduced to reconstitute bR layer on various substrates, including Langmuir–Blodgett deposition [77,78,79,80,81,82,83,84,85,86,87,88], electrophoretic sedimentation [85,89,90,91], biotin-streptavidin mediated monolayer [92,93], antibody-mediated monolayer [94,95] and electrostatic adsorption [22,96,97,98,99,100]. Figure 5 shows different typical PM preparation methods.

Corresponding to the PM preparation methods shown above, their characteristics are listed in Table 2; the charge difference between both sides of the bR molecule is the key point used in oriented bR layer preparation. Electric field, biotin-streptavidin binding interaction and electrostatic adsorption are diverse methods of using the spatial charge difference of the two sides. In these methods, antibody-mediated monolayer technique is good for deposition of PM monolayer. However, random orientation of the proceeding layers in antibody-mediated monolayer technique may result in reducing the functionality of the bR. By contrast, LB technique and electrophoretic sedimentation technique provide excellent control of PM orientation during film formation. Besides, electrodeposition is good for thick layers containing hundreds to thousands of PM layers, while electrostatic adsorption faces the difficulty in control of both orientation and layer numbers.

## 3. Photochemical Application of Bacteriorhodopsin

### 3.1. Optical Volumetric Memories

As described above, the bR protein translates proton orientation from the cytoplasmic side to the outside of the cell membrane, thereby converting the light energy into chemical energy. The proton transfer is accompanied by a series of spectral changes in the protein, known as the bR photocycle, which contains intermediate states with different spectral absorption maximums. In addition to the core loop, the bR cycle of the light has a branch cycle, from the O state to the P state to the Q state, and the P and Q states contain 9-cis-retinal in their photochromic groups. These states are achieved by photochemical excitation of all-trans-retinal, while Asp 85 is protonated simultaneously. Both proton transitive and 9-cis-conversion show low quantum efficiency. However, the consideration of branch states P and Q is critical in volume holography and page-oriented optical memory applications.

**Figure 6:**
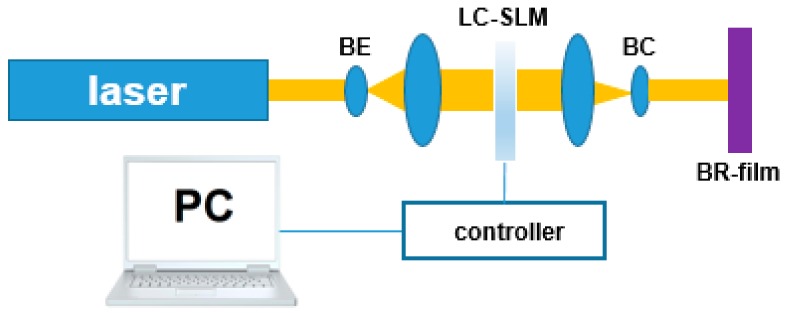
Setup for optical recording of data pages in bR films by two-photon absorption. Reproduced from [20].

**Figure 7:**
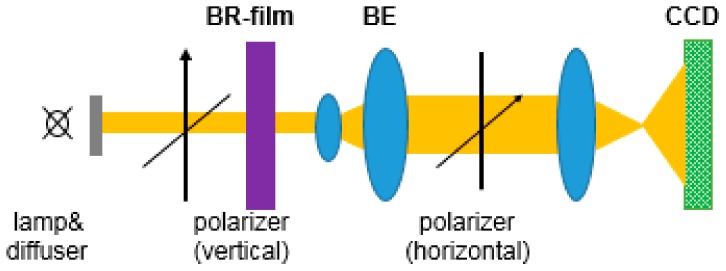
Optical setup for reading of data stored by employing permanent photo-induced anisotropy in bR films. Reproduced from [20].

The use of conversion from B to K state of bR protein can complete the data read and write. In 2003, the researchers proposed the use of bR as an active layer in a single write-in optical memory [20]. As shown in Figure 6, writing data to the memory area was done by pulsed-doubled Nd: YAG lasers (Coherent, Dieburg, Germany). The reading of the data stored in the bR film can be achieved by a conventional optical microscope or directly by a charge coupled device (CCD) matrix or a CCD line camera, as shown in Figure 7. The spatial distribution of photo anisotropy is easily observed by irradiating the bR film with a later polarized white light and observed by a rotatable linear polarizer. The recording of the information in the bR film was then performed by exposing the 3 ns pulses from the 532 nm light of the Nd: YAG laser. As an example of this new bR application, an identification card equipped with an optical record bar with a capacity of about 1 MB of data is provided. The current recording density is 125 kB/cm^2^, much lower than the optical limit, but allows the use of plastic optical devices for cheap operation.

**Figure 8:**
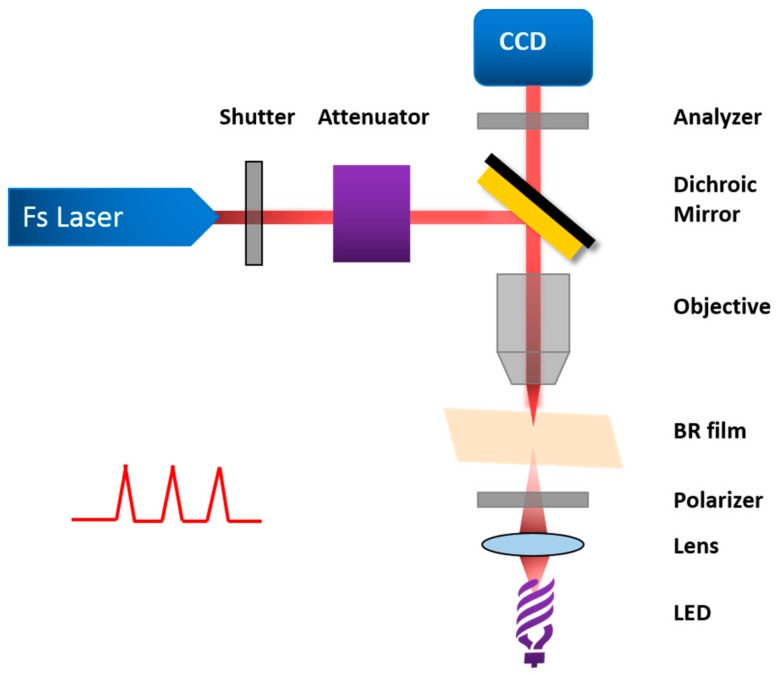
Optical settings for polarized multiplexing write once (WORM) optical memory. Reproduced from [22].

Most applications are based on the so-called M-type photochromic effect of bR, and the quantum efficiency is quite low, only about 0.02%, which limits its practical application and can only be used for transient optical data storage, while, in the case of bR, a light conversion product called F540 state excited by a 790 nm femtosecond laser pulse is stable for photochemical reactions or thermal pathways. Yao Baoli et al. studied the optical properties of the F540 state and analyzed the photo anisotropy of the F540 state using the Jones matrix theory [22]. When the bR film is excited by a linearly polarized femtosecond laser pulse, the selection of molecules occurs. Its long axis aligns the bR molecules in the light polarization direction and converts it into the F540 state, while the bR molecules in the direction perpendicular to the direction of polarization of the light are still in the B state. bR films exhibit strong anisotropic absorption after being excited by linearly polarized femtosecond lasers. However, in the case of excitation by the circularly polarized femtosecond laser beam, since the probability of the F540 state is equal in all directions, no photo anisotropy is observed in the experiment, and by using two polarization states of the femtosecond pulse laser, which demonstrates the permanent optical anisotropy of the write once read (WORM) optical data store (shown in Figure 8). Since the polarization information is also written on the storage medium, it is not possible to copy it in a common manner. This storage technology has potential applications in advanced optical security.

**Figure 9:**
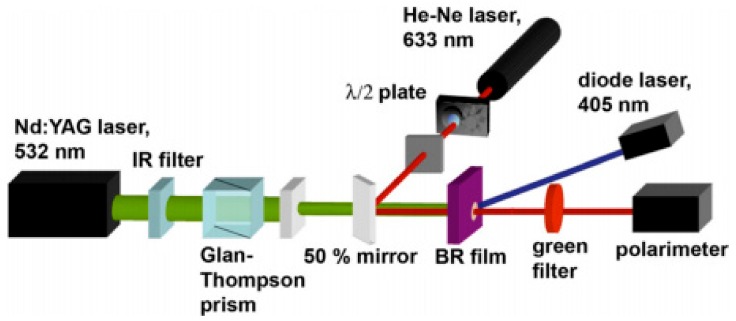
The linearly polarized 532 nm pulsed laser is used to cause permanent optical anisotropy in the bR film [21].

Besides the use of linearly polarized femtosecond laser, phase change of the light is also information for optical memories. Martin Imhof et al. measured the optical changes of the bR film caused by the TPA recording and determined the Muller matrix of the bR film [21], showing the potential application of bR film in safety technology. Researchers have demonstrated that the two-photon-induced photobleaching (TPP) converts the bR film to a phase mask so that it can use angle-multiplexing data storage in security technology. As shown in Figure 9, a readout is performed by a 632 nm tunable linearly polarized beam of a He-Ne laser using a frequency doubled Nd: YAG laser (Infinity 40–100, Coherent, k = 532 nm). The TPP of the bR membrane mainly leads to the phase change of the optical properties, and the absorption of the dichroism plays a very small role. Thus, the bR film is suitable for polarized data storage. bR film can not only carry optical information, but also act as a linear polarizer. In addition to its phase shift characteristics, a bR film containing data written with a linearly polarized laser beam is similar to a linear polarizer. This makes it possible to read the polarization data directly with only one polarizer.

As mentioned above, the bR protein has been used in the field of information storage for some preliminary applications. When the bR protein is excited by green light, it follows the light cycle and starts from the K state and ends with the O state. The second excitation caused by the red light raises the branching light cycle from O considered binary 0 to P and Q which is considered binary 1. During this P-state transition, the data are written and stored or read into the Q state. By using these binary files, the data are stored and read from the WH protein-based holographic record. The P and Q states are the height states that are capable of absorbing light in the blue spectrum. The stored data of the bR are erased by blue light excitation. In this way, it stores the perfect holographic material as a memory. bR not only allows writing and reading data, but also helps to erase, refresh and reset data. It refreshes every 1024 bits to complete once, and through the red light to continue to reset the lighting. bR protein could play an important role in future applications as a holographic material in CDMA, CNN-UM and POAC.

### 3.2. Holographic Associative Processors

**Figure 10:**
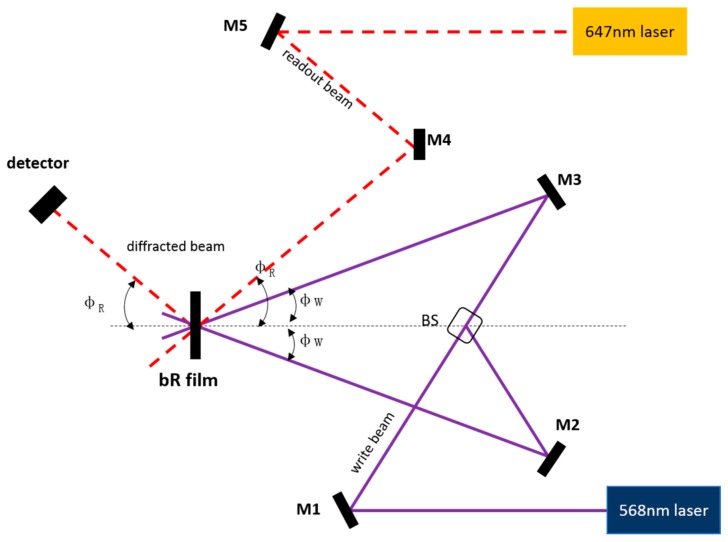
The light path diagram of the experimental set up applied in writing and reading volume transmission hologram. Reproduced from [23].

Bacteriorhodopsin has been serving as an archetypal biomaterial for studies involving optical processors. For example, from the view of computer science, Jordan A. Greco et al. explored the use of bacteriorhodopsin in optical associative processors recently.[23] They not only investigated complex Fourier association using computer simulations but also associated the results with the holographic properties of bR-based thin films and explored their uses in algorithms of processors. Owing to ability of bR-based thin film in combining dynamic photochromism with high quantum efficiency of the blue-shifted intermediates during the bR photocycle and structural stability, this work can provide a method for both real-time computing and long-term data storage. The light path diagram of the experimental set up applied in writing and reading a volume transmission hologram is shown in Figure 10.

Besides, from a biomolecule directed evolution standpoint, Jason. R Hillebrecht et al. investigated methods to optimize the photoactive protein bR for optical holographic memories recently [24]. They proposed optimization strategy of main and branched photocycle, key photochemical intermediates such as M-state, O-state, and Q-state using site-directed mutagenesis to improve efficacy of bR-based holographic memories. There have been over 800 semi-random mutants being studied for seeking the lengthened M-state and O-state lifetimes at 412 and 650 nm, respectively, through genetic modification. The length of lifetime M-state mutants can improve the holographic sensitive property of bR compared to the native bR for real-time holographic processors permanent. Another more permanent intermediate state, Q-state, is required for application of long-term holographic data storage. Amazingly, that device has the ability of regenerating the entire image by selecting from thousands of stored holograms only requiring a partial input one.

In conclusion, the first step towards achieving the goal of incorporating biomolecule into hybrid semiconductor-based holographic associative processors has been anticipated. With the process of technology, we have reason to believe that bR-based holographic associative processors have more potential foreground in both real-time computing and long-term data storage applications in the future.

## 4. Photoelectric Application of Bacteriorhodopsin

### 4.1. Motion Biosensors

Motion biosensor is a visual information system based on light-sensitive biomaterials, which instantaneously senses optical flow to detect the information of the motion of objects. Among biomaterials, bR is the most extensively studied. bR as a light-driven proton pump in nature can detect changes in the light just like human eyes, and it is the differential photocurrent response that reflects the information of moving objects. Motion biosensors are different from those traditional silicon-based AVLSI (Analog Very-Large-Scale-Integration) motion sensors. On the one hand, biomaterials are renewable and abundant, while, on the other hand, they are free of bias supply and need little time for preparation process.

**Figure 11:**
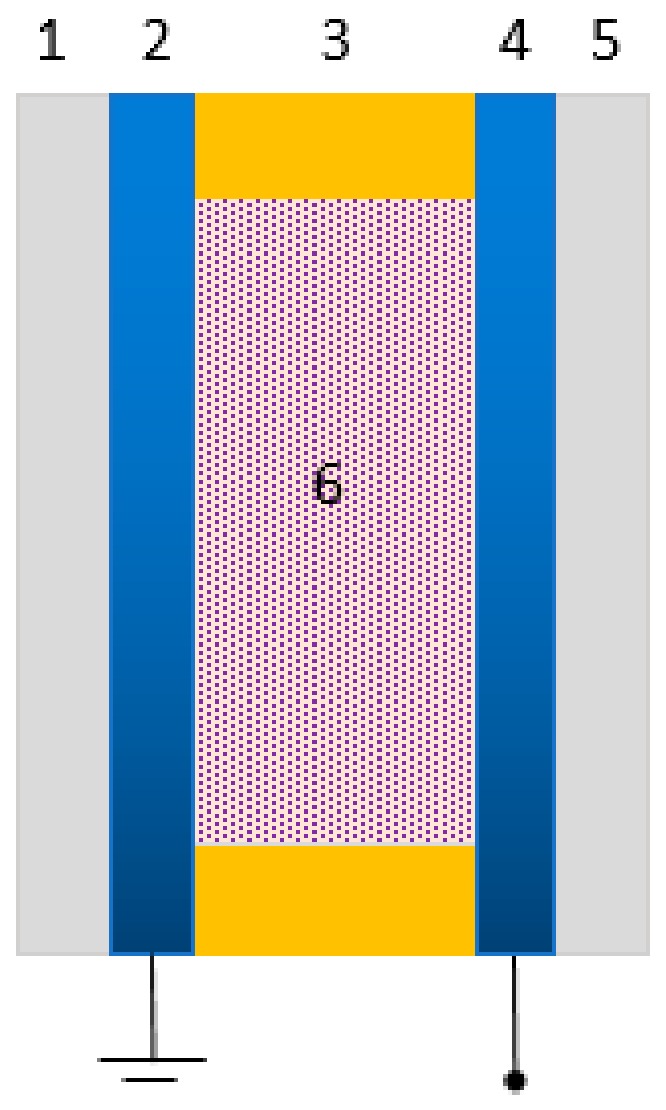
Structure of bR-based photocell. Sandwich structure: 1/5, glass substrate; 2/4, ITO transparent conductive coating; 3, polyester thin film; 6, deposited bR film. Reproduced from [27].

A high-speed motion detection system that utilizes light-sensitive bioelectronic components was explored in 2005 [27]. Each sensing element is a bR-based photocell which consists of a sandwich-structural, ITO (Indium Tin Oxide) electrode/bR film/ITO electrode, as shown in Figure 11. The photoelectric response of bR photodetector experimented by the current mode exhibits a wide dynamic range that is approximately linear over a light power range of μW to W and very fast response time up to μs. In addition, the device exhibits high-resolution because of the high degree of differential photosensitivity of bR material, which makes it possible to achieve real-time vision processing by a densely packed array of numerous bR photocells.

**Figure 12:**
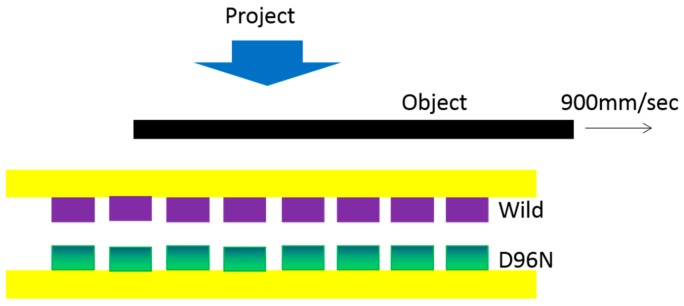
Experimental setup. A commercial projector was used as a light source. Reproduced from [25].

Over a long period of development of bR-based motion biosensor, there appears a novel construction of optical-flow sensing function using the bR biopolar photosensor array. For example, Yoshiko Okada-Shudo’s group developed a novel optical-flow sensing method [25]. They used a bipolar photosensor array with bR wildtype and its variant (D96N) and obtained the optical-flow curves of motion detection using a simple array. As shown in Figure 12, they deposited ITO in an 8 × 1 pixel pattern on a pair of substrates to fabricate the bipolar photosensor array and each pixel is square of 4 mm × 4 mm arrayed with a gap of 1 mm. To investigate further, research on a two-dimensional bipolar photosensor array with 8 × 8 pixels is under way.

**Figure 13:**
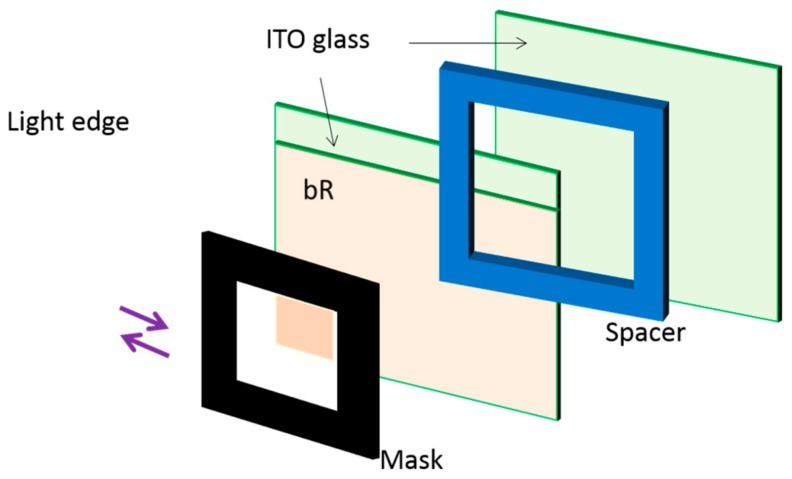
Structure of a bR-based photocell with a sensing pattern of square. The sandwich structure consists of a bR dip-coated film and electrolyte solution between two ITO glass. Reproduced from [26].

Besides, the single photocell marked with a pattern has been developed recently, which can detect the speed and direction of the moving light, especially can detect two or four directions owing to the asymmetry of the pattern. In another work, the group fabricated a photosynthesis protein-based sensor consisting of only one-sensing-element in free of complex software algorithms [26]. When light scans the patterned sensing area, the edges of each pattern can make photoelectric response, which shows information about light’s speed and direction as shown in Figure 13. They successfully detected the speed and directions even contain two or four of object motion owing to the asymmetry of those mask patterns. Besides, this motion sensor may be applied to contact-free gestural manipulation with a laser pointer for remote control systems.

Up to now, there have been various bR-based motion biosensors, from single sandwich-structural photocell to bR biopolar photosensor array or single photocell marked with a pattern, which may be applied to contact-free gestural manipulation. In general, bR-based motion biosensor has positive promise in remote control systems in the future.

### 4.2. X-ray Sensor

The technique of X-ray detection has still been under further development to improve its low sensitivity, complex processing requirements and so on. Therefore, some new materials have been used in the fabrication to achieve real-time and reusable radiation sensing recently. Bacteriorhodopsin, as a light driven proton pump protein molecule, has been used to fabricate photodetectors with sensing range from UV to infrared of the photospectrum since 2006 [100]. More importantly, in 2011, Ahmadi’s group proposed that bR could also be used in a sensor to detect the X-ray region for the first time and the flexible X-ray sensor based on bacteriorhodopsin they fabricated was able to achieve real-time monitoring of radiation dosage, energies and dose rates [35]. This flexible X-ray sensor is comprised of the following structure like a sandwich: PET-ITO-(bacteriorhodopsin, Kapton film)-ITO-PET. bR thin film is coated and dried on ITO electrodes which are connected to voltage supply. The Kapton Polyimide film is used to form an electrical insulation between two conductive ITO sheets. The sensing area is in the shape of a circular with radius of 3 mm.

As we know, X-ray interacts with matter in three main ways: photoelectric absorption, elastic and inelastic Compton scattering, and electron–hole pair production and these interactions will produce various types of electrons or photons. Which interaction mechanism it follows will mainly depends on the energy of the interacting X-ray and the composition of the matter. Because of changes in electric current that radiation induced, it can be used as a sensor to detect X-ray and its performance is evaluated under different dose rates, energies and field sizes. The output photocurrent is proportional to the dose rate and energy as well as field size increases. It also shows that integrated electric charge makes a linear response with the change of radiation dosage. In conclusion, the response of this flexible sensor could detect X-ray in real time. Before long, using bR sensor, Morteza Ahmadia’s group accomplished measuring X-ray beams in keV order in real time [102].

bR-based X-ray radiation detector can be readily miniaturized and relatively easy to fabricate and use. Besides, it possesses capability for real-time data collection and reusability. In a way, it has great potential in the military and medical field such as both diagnostic radiology and radiation therapy in the future.

### 4.3. Photovoltaic Cells

When the light stimulates on the surface of bR, there will be a series of photochemical reactions resulting in bR pumping protons from the intracellular side to extracellular side, which is the main reason for generation production of the photoelectric response.

In recent years, previous researchers have done several works on the application and mechanization of produced photocurrent. Studies on bR hybrid composite materials or sensors are based on inorganic and polymer materials under different operation mechanisms from enhanced proton transfer to electron transfer effect, as shown in Table 3.

**Figure 14:**
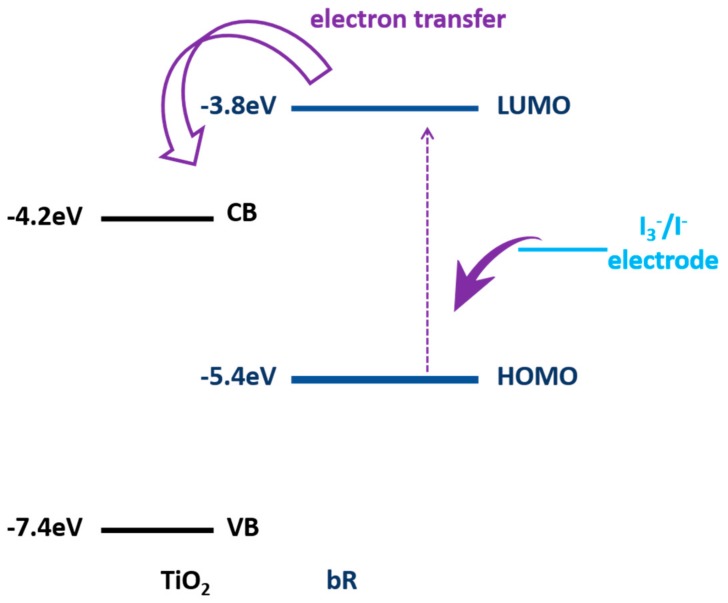
Energy level diagram and mechanism of carrier injection in the presence of redox electrolyte [28].

For example, an inorganic material, TiO_2_, was used as a composite material to construct a bR/TiO_2_ nanotube hybrid photoanode by anchoring bR molecules onto the surface of TiO_2_ nanotube arrays by Nageh K. Allam’s group [28]. The bR/TiO_2_ nanotube hybrid photoanode showed an enhanced photocurrent performance compared to either TiO_2_ or bR alone. That is because bR could provide another proton production channel once bR is anchored to the surface of TiO_2_ and the electrons from the redox molecules could be caught by bR and injected into TiO_2_ surface in turn in the assistance of redox electrolyte, as shown in Figure 14. This bR/TiO_2_ hybrid photoanaode shows a respective direction for development of bio-photovaltage cells.

**Figure 15:**
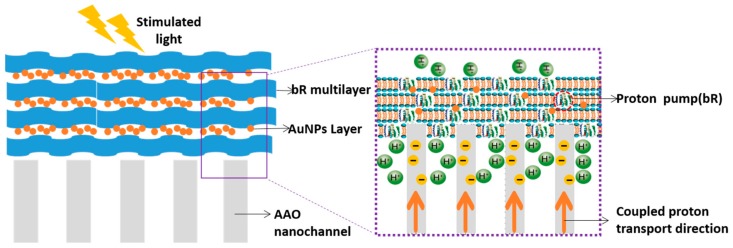
Scheme of the bR/AuNPs heterogeneous structure with bR and AuNPs deposited on the AAO nanochanels. The protons were transferred from CP side to EC side when light illuminates, and the photocycle path was shortened with the assistance of surface plasmonic effect of AuNPs. Reproduced from [29].

Introducing inorganic materials to bR as such for enhancing the photoelectric properties of hybrid structures is not rare. Except TiO_2_, Zhibin Guo et al. firstly illustrated a novel photovoltaic stacking system with the structure of bR/AuNPs heterogeneous multilayers with improved photoelectric performance, as shown in Figure 15 [29]. With this stacking system, photoelectric performance was improved effectively owing to the cooperative control of proper diameter of AuNPs and the stacking layers. It can modulate input flickering light into regular photoelectric signals to be well controlled. In a degree, this system possesses potential foreground in solar energy converter to power.

**Figure 16:**
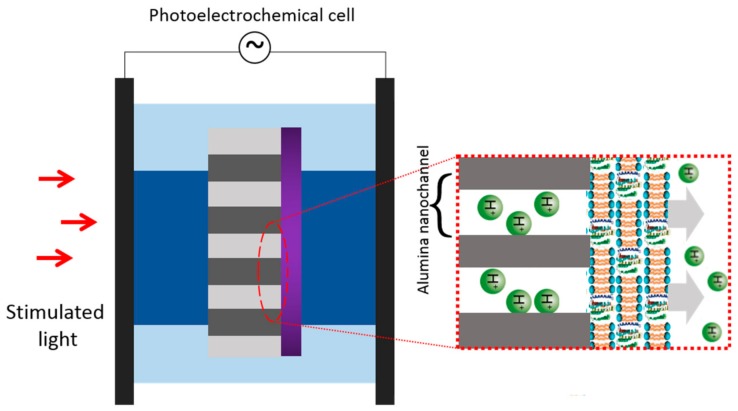
Schematic illustration of the photoelectrochemical cell. Oriented bR multilayers were deposited onto alumina nanochannel arrays by electrophoretical method and the CP side of bR faced to the alumina membrane side. Reproduced from [30].

For the study on photocurrent signals of bR, it is worth paying attention to the frequency-responsive characteristic besides photocurrent amplitude, which helps in mimicking of mammalian retina. Shanfu Lu et al. constructed a microstructure of bacteriorhodopsin/alumina nanochannel as shown in Figure 16 [30]. With this hybrid photoelectric nanosystem, flickering light impulses can be converted into distinguishable photocurrent in high frequency up to 130 Hz. They achieved mimicking frequency-responsive characteristic of in vitro mammalian retina. The proton concentration gradient, the thickness of bR multilayers and the pore size of alumina nanochannel are all factors in regulating the photocurrent. This hybrid system will have a broad and prospective application for artificial retina.

**Figure 17:**
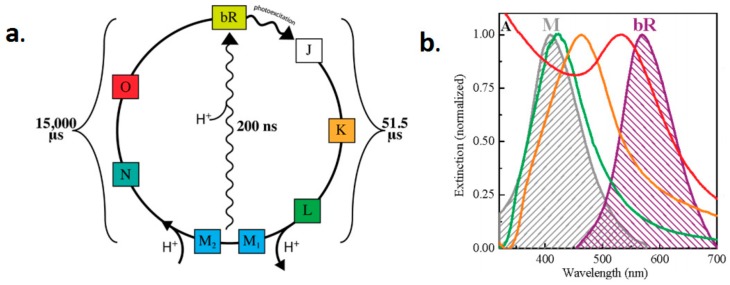
(**a**) Photocycle of bR; and (**b**) Normalized Surface Plasmon Resonance spectra of Ag NPs (425 nm in green), Ag-Au NPs (465 nm in orange) and Au NPs (530 nm in red). Gray and violet shadows represented the absorption of M412 state and bR570 state, with maxima corresponding 412 and 570 nm respectively [33].

In addition to these applications, it is also worth considering the mechanism of the influence of bR photocycle on photocurrent. Blue light effect is an interesting phenomenon that has been extensively studied. Specifically, the long-lived intermediate M_412_ can be generated in 70 μs and revert to ground state, bR_570_, thermally through many other intermediates in 15 ms. However, except for the traditional way, there is another rapid method through the photochemical process. It excited blue light in hundreds of nanoseconds so it is called blue light effect. Chun-wan Yen et al. found this surface plasmon enhancement effect of the M_412_ absorption can not only increase the rate of proton release but also force bR follow the photocycle of short time bypassed path that exciting M_412_ to form bR_570_ rapidly compared to the conventional thermal one, as shown in Figure 17 [33,34]. They explored the photocurrent density of mixing bR with various nanoparticles, such as Ag NPs, Ag-Au alloy NPs and Au NPs with different concentration and pore diameter.

**Figure 18:**
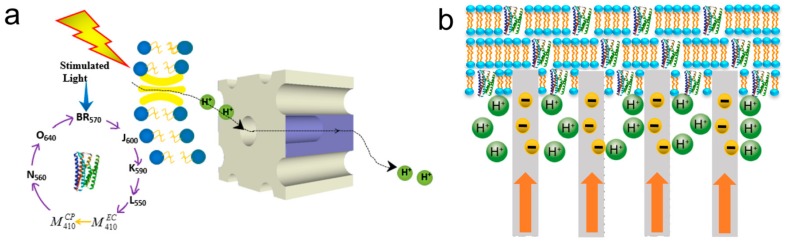
Schematic diagram of two methods to export optical signals of bR. (**a**) Creating a photocell by producing the proton gradient difference between the two half-cell. (**b**) The proton coupled with carrier, inducing charged carrier displacement and recombination during the bR photocycle, generating a differential photovoltage.

There are two methods to export optical signals of bR (shown in Figure 18). One is to create a photocell, producing the proton gradient difference between the two half-cell. The other is the proton coupling with carrier, inducing charged carrier displacement and recombination during the bR photocycle, generating a differential photovoltage. The research now mainly focuses on the use of hybrid structures to enhance photocurrent or frequency response. We are very optimistic that photocurrent performance of bR is expected to be used in the field of biological electronics and will possess potential applications in medical electronics.

### 4.4. bR Related Immunosensor

With the development of bR, more and more new applications have emerged, and, in 2017, its photoelectric characteristics made a breakthrough in the field of microbial detection.

A bR-based photoelectric immunosensor was first demonstrated for direct, label-free, selective, and sensitive microbial detection by Hsiu-Mei Chen et al. [37]. Antibodies are affinity-immobilized on purple-membrane coated electrode and the photocurrent generated by the antibody-coated sensor was reduced after incubation with *E. coli* K-12 cultures, with the reduction level increased with the culture populations. The photoelectric immunosensor could achieve the quantitative single-step detection up to 1–10^7^ CFU/10 mL microbial cultures. To explain the detection principle, they investigated the effect of illumination orientation and simulated the photocurrent responses with an equivalent circuit model containing a chemical capacitance, and, finally, they considered the bacterial light-shielding effect as the reason for photocurrent reduction.

Using current fabrication technique, we believe that versatile bR-based photoelectric immunosensors will have a wide and promising future in the field of biological cells detection.

### 4.5. bR Related Artificial Retinal Prostheses

With more and more attention on bioelectronics for medical care recently, bacteriorhodopsin has been explored as an important photoactive element in the protein-based retinal prosthesis used for patients suffering from retinal degenerative diseases.

**Figure 19:**
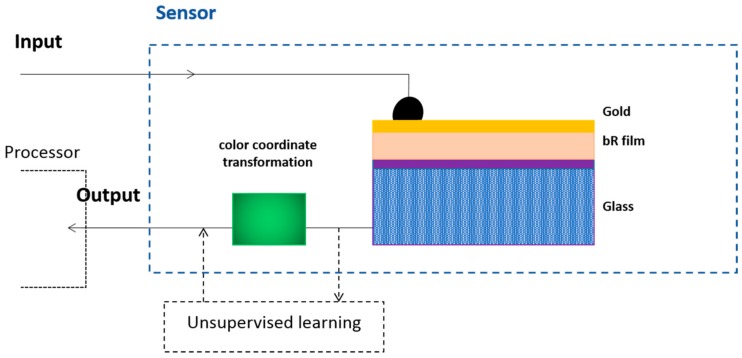
The system of bR-based artificial retina and the scheme of the bR element. The 40 nm thick gold layer is on the mixed film of PVA and bR as electrode of the conductive glass. Reproduced from [19].

Michael Frydrych et al. produced a simple color-sensitive artificial retina, in which bR and its two artificial variants were used to form a matrix as photosensitive elements [19]. The outstanding advantage of this retina was its easy construction and high performance in recognition of color, achieving low-level processing of input information in color space transformation to improve the spatial resolution. They employed an artificial neural network in the system to learn self-organizing color adaptation algorithm to tune color space transformation parameters to learn colors and adapt to an environment as a natural system does. In this system, the optoelectrical signal generated by the elements consisting of three proteins was amplified and connected to a computer that simulated as the preprocessing layer as shown in Figure 19. Owing to bR whose photochromic or photoelectric properties were employed in this system belonged to biomaterials, the system was regarded as an intelligent color-sensitive artificial retina.

The use of orientated bR in generating the photoelectrical signal can show its unique performance of rapid responsivity, high quantum efficiency. Besides, it possesses optical potentials in coupling its response with charge-sensitive semiconductor arrays directly. Thus, it has been widely used as a photosensitive element in artificial protein-based retinal prosthesis. Except that, artificial retinas based on bR exhibit excellent ability of differential responsivity, edge enhancement and motion detection. Based on the above, we have full reason to believe that bR will have an enormous breakthrough in the study of artificial retinal prostheses in the future.

## 5. Conclusions/Outlook

This article provided a review of recent progress in bR-based bioelectronic devices. The proton pump function and specific photocycle intermediates of bR molecular have lead to many photochemical and photoelectric applications, which contribute greatly to the creation of new biosensors and bioelectronics. For the photochemical applications, optical volumetric memories perform well in optical information storage by using long-life Q states, while holographic associative processors have more potential foreground in both real-time computing and long-term data storage applications. As for the photoelectric application, motion biosensors may be applied in contact-free gestural manipulation, and photovoltaic cells are most investigated by the method of proton gradient difference or proton coupling with carrier. Besides, bR related artificial retinal prostheses exhibit excellent ability of differential responsivity, edge enhancement and motion detection. It is worth noting that not all bR-based bioelectronic applications mentioned above are still being researched. Research status of these bR-based bioelectronic applications is shown in Table 4, leading to a clear research direction for future research on bR-based bioelectronic device.

The brief summary of these applications demonstrated in the article are sufficient to generalize the relatively new bR-based biotechnology and inspire researchers to design new high-performance bR-based bioelectronic devices.

## Figures and Tables

**Table 1:** Comparison between bR and other similar proteins in *H. salinarum.*

Protein	Ion	Sensitive Light Color	Function	Contents
bR	H^+^	Green	Energy conversion	2,000,000/bacteria
hR	Cl^−^	Green	Maintain osmolarity	20,000/bacteria
sR-I	H^+^	Blue/Green	Avoid blue light	5000/bacteria
sR-II	H^+^	purple	Avoid purple light	5000/bacteria

**Table 2:** Characteristics of different PM preparation methods.

Methods	Orientation	Number of Layer	References
Langmuir–Blodgett deposition	High degree of control	Excellent control over layer numbers	[77,78,79,80,81,82,83,84,85,86,87,88]
Electrophoretic sedimentation	High degree of control	Multilayer	[85,89,90,91]
Biotin-streptavidin mediated	High degree of control	Monolayer	[92,93]
Antibody-mediated monolayer	High degree of control	Monolayer	[94,95]
Electrostatic adsorption	Difficult to confirm	<12	[96,97,98,99]

**Table 3:** Operation mechanisms of previous bR-based devices.

Composite Materials	Operation Mechanisms	Literature
bR/TiO_2_	Electron transfer effect	Energy Environ. Sci., 2011 [28]
bR/Au	Coupled proton transportation	Nano Energy, 2015 [29]
pR/kpw	3D Proton transfer	Adv. Mater., 2015 [32]
bR/AAO	Protons transportation	Adv. Mater., 2016 [30]
bR/Nafion	Plasmonic enhancement	Nano letters, 2011 [34]
bR/QDs	Förster resonance energy transfer	ACS nano, 2013 [31]
bR/CNTs	Electron–ion interaction	Nanoscale, 2018 [103]

The abbreviations kpw, AAO, QDs, and CNTs mean potassium phosphotungstate, anodized aluminum, quantum dots, and carbon nanotubes, respectively.

**Table 4:** Research status of all bR-based bioelectronic application.

Application Direction	Application Category	Currently Being Researched	Reason for Stop
Photochemical application	Optical volumetric memories	Yes	—
	Holographic associative processors	No	Poor compatibility and replaced by new optical information recording materials
Photoelectric application	Motion biosensors	No	Expensive material and weaker electrical signal
	X-ray sensor	Yes	—
	Photovoltaic cells	Yes	—
	Immunosensor	Yes	—
	Artificial retinal prostheses	No	Security and human biological compatibility
